# Schoolteachers’ Knowledge of Attention-Deficit/Hyperactivity Disorder—Current Status and Effectiveness of Knowledge Improvement Program: A Randomized Controlled Trial

**DOI:** 10.3390/ijerph17155605

**Published:** 2020-08-03

**Authors:** Abdullah M. Alshehri, Shehata F. Shehata, Khalid M. Almosa, Nabil J. Awadalla

**Affiliations:** 1 Joint Program of Saudi Board in Community Medicine, Southern Region 61421, Saudi Arabia; amd.a1411@hotmail.com; 2 Department of Family and Community Medicine, College of Medicine, King Khalid University, Abha 61421, Saudi Arabia; shehatafarag@yahoo.com; 3 Department of Biostatistics, High Institute of Public Health, Alexandria University, Alexandria 21511, Egypt; 4 Community Medicine Consultant, Joint Program of Saudi Board in Community Medicine, Southern Region 61421, Saudi Arabia; drkalmosa@hotmail.com; 5 Department of Community Medicine, College of Medicine, Mansoura University, Mansoura 35516, Egypt

**Keywords:** attention-deficit/hyperactivity disorder, ADHD, teachers, educational program intervention, Saudi Arabia

## Abstract

Background: Adequately knowledgeable schoolteachers can play an essential role in early detection and proper treatment of children with attention-deficit/hyperactivity disorder (ADHD) at school. Objectives: To assess the schoolteachers’ knowledge of ADHD and to evaluate the effectiveness of the ADHD knowledge improvement program. Methods: A randomized controlled trial study design was followed on 100 primary school teachers from Abha City, Saudi Arabia. Teachers were randomly selected and allocated into trial and control groups. A self-administered questionnaire was used to evaluate teachers’ knowledge about ADHD. After the baseline assessment, participants in the trial group received a two-day ADHD knowledge improvement program. Teachers’ knowledge about ADHD was reassessed using the same questionnaire immediately after finishing the intervention program and after three months in both groups. Results: Only 16% and 22% of trial and control groups, respectively, showed adequate baseline knowledge about ADHD. Teachers’ main sources of information about ADHD were the internet (67%), friends (47%), TV (34%), and reading books (23%). Knowledge of teachers in the trial group significantly improved immediately after the intervention. After three months, these knowledge benefits slightly declined but remained more adequate than those of teachers in the control group. The logistic regression model revealed that being a participant in the trial group and of higher qualification were associated with significant improvement in teachers’ retained knowledge on ADHD (*p* < 0.001 and *p* = 0.050, respectively). Conclusions: The majority of male primary school teachers in Abha City, Saudi Arabia, have inadequate knowledge about ADHD. Applying a knowledge improvement program can substantially improve their knowledge of ADHD. Consideration should be given to the integration of ADHD knowledge improvement program into teachers’ educational programs.

## 1. Introduction

Attention-deficit/hyperactivity disorder (ADHD) is one of the most common chronic mental disorders that can affect school-aged children. It is characterized by a persistent pattern of impulsivity, hyperactivity, and inattention problems [1,2]. ADHD is usually occurring in early childhood and may continue into adulthood causing academic underachievement and reduced self-esteem [3]. It is usually associated with learning disabilities and comorbid behavioral disorders, which can impede the successful development of the affected children [4]. Children with ADHD are more likely than peers to develop conduct disorder and antisocial personality disorder in adulthood, consequently increasing the risk of substance use disorders and incarceration [2].

Worldwide, the prevalence rates of ADHD ranged from 2.2% to 17.8% [5]. A more recent systematic review study in Arab countries reported the prevalence of ADHD ranged between 1.3–16% [6]. To help children having ADHD to cope with their symptoms, they need continuous assistance from family, and adequately knowledgeable schoolteachers and counselors [7].

Primary school teachers play an essential role in the assessment of children’s behaviors and can be the first ones to identify children with ADHD [8]. Additionally, teachers are necessary for implementing and evaluating the ADHD treatment plan for school children [9].

Different questionnaires and self-reports are available to assess teachers’ impressions of a potential student with ADHD. The most common tool used was Conners’ rating scales [10]. In Saudi Arabia, there are indications that teachers may not have enough knowledge about ADHD [11,12]. This issue may increase the risk of delayed diagnosis, misdiagnosis or improper school treatment of children with ADHD [12,13]. A multinational study on teachers from nine countries indicated that professional ADHD training predicted adequate knowledge in the majority of countries [14].

Recently, some tools invented to help schoolteachers in the diagnosis and behavior management of students having ADHD. Teachers should be aware of their existence, as well as their usefulness. These tools include Aula Nesplora in primary schools [15] and Aquarium Nesplora in secondary schools [16].

Several studies have recommended educational program intervention to improve teachers’ knowledge about ADHD and consequently their practice with children having ADHD [3,12,17,18]. A recent study in Saudi Arabia highlighted the need to professional training to improve teachers’ capabilities to diagnose and treat children with ADHD [19]. However, information regarding the effectiveness of the education intervention in Saudi Arabia is scarce. 

The objectives of the present study were to assess the schoolteachers’ knowledge regarding ADHD and to evaluate the effectiveness of the ADHD knowledge improvement program among male primary school teachers’ in Abha City, Saudi Arabia.

## 2. Methods

### 2.1. Study Design and Setting

The present study is quantitative community-based research. It followed a randomized controlled trial research design and carried out during the period from September 2019 to January 2020 on governmental and private male primary schools in Abha city. Abha city is the capital of the Aseer region, which located in the southwest of Saudi Arabia. It incorporated 51 public schools and 12 private schools with a total of 1341 male teachers: 1091 in public schools, and 250 in private schools.

### 2.2. Sample Size Calculation and Sampling Technique

The target population was male teachers at primary schools in Abha city. The sample size was calculated based on Zhong [20], with the estimated average effect size for intervention efficacy being not less than 0.9 (large) [21], a variance ratio of 1.8, and a design effect of 2, at 95% confidence level, with an allocation ratio of 1:1 and dropout rate of 10% that provide a study power of 85%. A total of 100 teachers (50 teachers per study group) were recruited in this study. Teachers previously exposed to any educational or training programs on ADHD and those not directly involved in the teaching process were excluded.

A stratified two-stage cluster sampling technique was used. Firstly, schools were stratified by type of schools into public and private. Then, eight public schools and two private schools were selected by a simple random technique. Next, from each school, the teachers were enlisted, and 10 male teachers were selected by a systematic random sampling method from each school. None of the schools involved in the study has school counseling services.

### 2.3. Random Allocation

To avoid contamination, the selected schools were randomly allocated to either the trial group or control group using computer-based randomization. Each group included 50 teachers from four public primary schools and one private school. 

### 2.4. Study Tools and Data Collection

A self-administered questionnaire, which was developed by Awadalla et al. [10], was used to evaluate teachers’ knowledge about ADHD. This questionnaire was in the Arabic language and it has a content validity index amounted to 0.97 and alpha Cronbach’s value of 0.79. The questionnaire is composed of 6 items covering socio-demographic characteristics of teachers (age, nationality, years of experience, qualification, specialty, and type of school) and 20 items assessing teachers’ knowledge of ADHD. Each knowledge item was assessed on a 2-point scale (incorrect response or do not know = 0, and correct response = 1), with a maximum score of 20. The questionnaire ADHD knowledge items comprised the following: nature of disease (1 item), age of onset, sex prevalence, disease course, symptoms (7 items), risk factors (4 items), aggravating factors (4 items) and management. Adequate knowledge was considered when a participating teacher achieved 65% of the total score. The content of the questionnaire was validated by ADHD experts rating each item.

The Data collection tool was used to assess socio-demographic characteristics and the baseline knowledge about ADHD for both experimental and control groups. After the baseline assessment, participants in the trial group received a two-day ADHD knowledge improvement program. Teachers’ knowledge about ADHD was reassessed in both groups using the same questionnaire immediately after finishing the program sessions and after three months interval to detect retained knowledge.

### 2.5. Teachers’ Knowledge Improvement Program

The program was developed by the first author after reviewing the current literature, the World Health Organization’s Mental Health Gap Action Program Intervention Guide (MhGAP-IG) [22] and the Saudi ADHD Society online teacher toolkit [23]. The program was delivered to the teachers in the trial group by the first author. The program objectives were to emphasize the significance and burden of ADHD, develop the capability of primary school teachers for early recognition, and improve the classroom treatment of ADHD.

It was conducted during a two-day workshop. On the first day, participants attended a one-hour lecture about ADHD covering the following: an overview of ADHD; the prevalence in Saudi Arabia; causes, symptoms and risk factors; associated impairment and prognosis; different treatment approaches, including behavioral interventions and medication. This lecture was done using Microsoft PowerPoint with audiovisual aids. At the end of the session, open discussion with the participants was done to answer there inquires. A hard copy of the lecture was given to the participants and the school director. On the second day, they attended a workshop about the teachers’ toolkit for ADHD, which contained how to identify ADHD and strategies for dealing with ADHD students in the classroom. Brochures about this disorder were distributed at the end of the program. Teachers attended the program in their schools using the library or the learning resource room with a maximum of ten teachers for each session. Educational materials were offered to the control group after the third assessment.

### 2.6. Statistical Analysis 

Data entry and analysis were performed using the Statistical Package for Social Sciences (IBM-SPSS, version 23). Quantitative variables were presented as means and standard deviations, while categorical variables were presented as frequencies and percentages. Chi-square test and Friedman test were applied to assess differences between the trial and control groups. The Mann–Whitney U test was used to compare the overall knowledge score of trial and control groups at different points of assessment. Hierarchical multivariate logistic regression analysis was used to assess the individual effect of intervention after controlling all co-variance. The dependent variable was teachers’ knowledge at the third month post intervention, and the independent variables were: study group (trial vs. control), age (less than 40 years old vs. above 40 years), nationality (non-Saudi vs. Saudi), qualification (postgraduate vs. university), experience (above 10 years vs. below), school (private vs. public). *p*-values < 0.05 were considered as statistically significant.

### 2.7. Ethical Considerations

The research proposal was submitted and approved by the Research Ethical Committee in General Directorate of Health Affairs, Aseer Region (REC-NO:2-8-2019). An official approval was obtained from the Directorate of Education in Abha city and the schools’ authorities. An informed consent was obtained from all participant before data collection. The informed consent included their voluntary participation, the right to withdraw from the study at any time without penalty or loss of any benefit from the institute/organization and assured confidentiality and privacy of participants.

## 3. Results

### 3.1. Participants’ Characteristics

The present study included 100 male primary school teachers selected from eight public schools and two private schools (10 teachers from each school). They were randomly allocated into trial and control groups (four public and one private schools in each group). To minimize the possibility of contamination, different schools were assigned for trial and control groups. Most of the teachers (51%) were less than 40 years old, Saudi (81%), having a university degree (86%), and 10–19 years of experience (48%). All of them have a Bachelor of Education and 14% have a postgraduate degree. They represented different teaching classes at primary schools. Most of them teaching Arabic (24%), religion (21%), and mathematics (17%) courses. There were no statistically significant differences between the trial and the control groups regarding participant’ characteristics. All participants did not previously receive any training about ADHD.

All teachers in both groups were aware about ADHD. The teachers’ main sources of information about ADHD were the internet (67%), their friends (47%) and the television (34%). Books were the sources of information for only 23%.

### 3.2. Teachers’ ADHD Baseline Knowledge

According to Table 1, only 16% and 22% of trial and control groups, respectively, had adequate knowledge about ADHD (achieved ≥65% of the total score). Interestingly, the lowest correct responses were reported for causes and aggravating factors. No statistically significant differences were found between both study groups (*p* > 0.05).

### 3.3. Effectiveness of ADHD Knowledge Improving Program

Based on Table 1, the proportion of correct responses to all teachers’ knowledge items about ADHD items differed significantly across the three phases in the trial group, with a marked increase in the proportion of correct responses immediately after the intervention, followed by a slight decline in the proportion of correct responses after three months. Regarding teachers’ knowledge about ADHD items in the control group, there were no significant variations in the proportion of correct responses over the different assessment points (*p* > 0.05). However, significant improvements were detected only in two items, i.e., the progress of the disease and treatment lines (*p* = 0.022 and *p* = 0.014, respectively).

**Figure 1:**
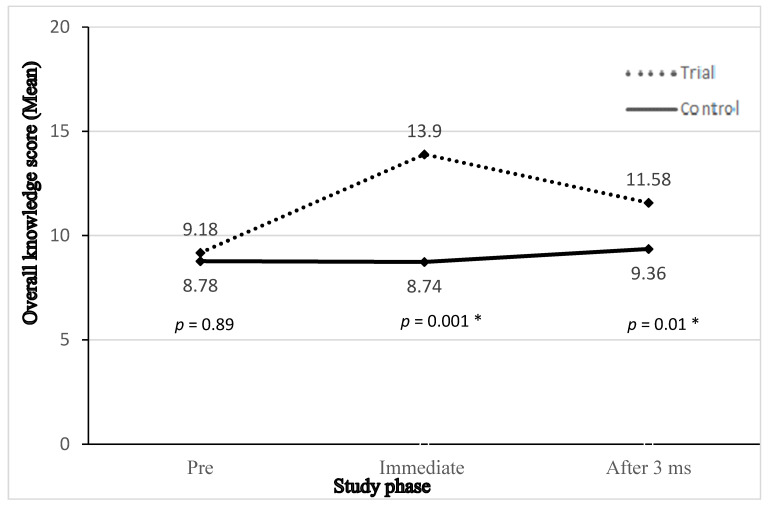
Teachers’ overall knowledge score in trial and control groups at different points of assessment. * Statistically significant (*p* < 0.05).

Regarding the overall knowledge score, immediately after the intervention, the overall knowledge of teachers in the trial group became more adequate than that in the control group (70% and 20%, respectively). After three months, knowledge of teachers in the trial group remained more acceptable than that in the control group (46% and 28%, respectively). Furthermore, Figure 1 shows that the average overall knowledge score for teachers in the intervention group pursued a marked immediate increase after the intervention, followed by a slight decline after three months. On the other hand, the average overall knowledge score for teachers in the control group remained almost the same across different study phases. Significant differences between both groups were observed immediately after the intervention (*p* = 0.001) and after three months (*p* = 0.01).

Result of multiple hierarchical logistic regression model shown in Table 2 revealed that being a participant in the intervention group and having higher qualification were associated with significant improvement of teachers’ knowledge level regarding ADHD (*p* < 0.001 and *p* = 0.040, respectively).

## 4. Discussion

Teachers spend a long time at school with their students. They are usually aware of students’ behaviors in different classroom situations. Therefore, they expected to be the first to observe and detect ADHD among their schoolchildren [24]. Unfortunately, our study revealed insufficient knowledge about different aspects of ADHD among male primary school teachers in southwest Saudi Arabia. Only 16% and 22% of trial and control groups, respectively, had adequate knowledge about ADHD. Similarly, several studies found that teachers’ knowledge of ADHD is insufficient [11,12,25]. This issue may increase the possibility of delayed diagnosis of ADHD, classroom mistreatment, and may worsen the consequences and prognosis of the condition [12,24,26,27]. Inadequate teachers’ knowledge can also lead to the spread of misconceptions [28], gender-bias [29], inappropriate interventions [30], and use of improper punishment techniques [25].

The present study revealed that the most important sources of ADHD information, in order, were as follows: internet, friends, television, and books. Similar results were reported in Riyadh, Saudi Arabia [8], and in Qatar [31]. Acquiring knowledge of health-related issues through the internet and TV may carry risks of information being misunderstood, resulting in misconceptions or misbehaviors [11]. Developing reliable and scientific online sources of ADHD is crucial for the teachers to protect them from being exposed to inaccurate and unreliable sources of information [32].

The baseline status of teachers’ knowledge regarding ADHD in the present study confirms the need for effective schoolteachers’ intervention [14]. The current study examined the effectiveness of a school-based teachers’ ADHD knowledge improvement program before its use at a national level in Saudi Arabia.

The results of our study showed that the overall knowledge of teachers in the trial group about ADHD was significantly improved immediately after the intervention. Then, these knowledge benefits were slightly declined but could last for three months and remained more adequate than those in the control group. The substantial improvement in teachers’ knowledge about ADHD following the intervention in the present study is in agreement with the findings reported by several studies using educational or training methods, which have all shown rapid improvement in teachers’ knowledge about ADHD, with benefits remaining for up to 6 months [21,33,34,35]. A previous study compared the effectiveness of two different training methods on ADHD among primary school teachers—namely, a two-day workshop and nonattendance education by ADHD booklets. Both training methods were effective in improving teachers’ knowledge of ADH. Yet, the two-day training was more effective in improving teachers’ attitude and practice [36].

In comparison with previous interventional studies where training has to proceed throughout several days [33] and by three tutors [33,35], the present two-day workshop intervention is more efficient in improving teachers’ knowledge (less time spent, one tutor and utilizes the existing school resources).

The results of the multivariate logistic regression model revealed that attending the educational program on ADHD was the most significant predictor for teachers’ post-intervention retained knowledge about ADHD. The two-day face-to-face workshop implemented in the present study is effective in improving ADHD knowledge among attended teachers. However, the observed decline in the retained knowledge score at three months among the trial group highlights the need for periodic reminder training [37]. Updating the training materials according to the new ADHD guidelines in the revision workshops will be very helpful.

The multivariate logistic regression model revealed that having postgraduate qualification was associated with significant improvement of teachers’ retained knowledge. These results are in accordance with findings of previous research from Saudi Arabia, which have consistently shown more qualified teachers and those who received educational programs on ADHD had more adequate knowledge about ADHD [8,38]. These studies also recommended to include ADHD training and educational programs into the postgraduate curricula of teachers, with repeated enforcement through in-service training.

Although acquiring enough knowledge about ADHD is necessary to have appropriate an attitude and proper practice [37], the present study cannot assure attitude and practice development in real situations. Real situation analysis of teachers’ performance may be important to confirm the program’s effectiveness regarding this issue.

## 5. Study Limitations

Although the present study confirms the effectiveness of the intervention program in improving teachers’ knowledge. Few limitations should be considered in interpreting our results. Firstly, due to strict time constraints, the duration of the intervention and follow up was only three months. Therefore, the long-term impact of the applied knowledge improvement program could not be assessed. However, the decline of retained knowledge in the third month may indicate the need to renew training and long-term impact assessment. Secondly, we used a knowledge assessment tool to explore the program’s effectiveness. An on-site practice assessment may be also necessary to confirm teachers’ performance improvement. Another limitation in questionnaire coding, we combined both incorrect and do not know responses together and gave them the same score. This issue hardens the distinction between teachers’ misconceptions and lack of knowledge. Finally, the study involved only male primary school teachers in Abha city. This issue may minimize the generalizability of our results.

## 6. Conclusions

The study highlighted the inadequacy of primary school teachers’ knowledge about ADHD in Abha city, Saudi Arabia. A two-day ADHD education program is effective in improving teachers’ knowledge. Consideration should be given for integrating the ADHD knowledge improvement program into teachers’ educational and training programs. This program should be part of their continuous professional development. Periodic reminder training is suggested to update and refresh their knowledge. Field assessment may be necessary to confirm teachers’ practice improvement. Further studies are needed in various regions of Saudi Arabia, with a longer follow-up duration to evaluate the long-term impact of the program. Building up trustworthy ADHD online resources is recommended to improve teachers’ capacity and to protect them from unreliable information.

## Figures and Tables

**Table 1:** Proportion of teachers’ correct responses to ADHD knowledge assessment questionnaire in the three study phases in trial and control groups (*n* = 100).

Knowledge Items	Trial Group			*p*-Value ^a^	Control Group			*p*-Value ^a^
	Pre-Training	Immediate	3 Months		Pre-Training	Immediate	3 Months	
	No. (%)	No. (%)	No. (%)		No. (%)	No. (%)	No. (%)	
General knowledge Nature of ADHD	33 (66)	48 (96)	48 (96)	0.001 *	32 (64)	34 (68)	36 (72)	0.692
Age of onset	35 (70)	47 (94)	43 (86)	0.005 *	34 (68)	35 (70)	37 (74)	0.798
Progress	16 (32)	32 (64)	26 (52)	0.005 *	14 (28)	15 (30)	26 (52)	0.022 *
Sex prevalence	31 (62)	42 (84)	40 (80)	0.025 *	31 (62)	28 (56)	35 (70)	0.348
ADHD symptoms Child is easily distracted	35 (70)	45 (90)	45 (90)	0.008 *	29 (58)	31 (62)	34 (68)	0.582
Child has trouble awaiting his turn	37 (74)	45 (90)	38 (76)	0.045 *	28 (56)	30 (60)	30 (60)	0.896
Does not seem to listen when spoken to directly	30 (60)	37 (74)	27 (54)	0.105	28 (56)	26 (52)	26 (52)	0.878
Often fidgets with hands or feet	38 (76)	45 (90)	44 (88)	0.11	34 (68)	30 (60)	32 (64)	0.707
Often talks excessively	20 (40)	30 (60)	21 (42)	0.044 *	22 (44)	23 (46)	24 (48)	0.923
Often gets up from seat when remaining in seat is expected	38 (76)	44 (88)	37 (74)	0.144	34 (68)	29 (58)	30 (60)	0.552
Often performs life-threatening acts	25 (50)	37 (74)	36 (72)	0.020 *	27 (54)	25 (50)	20 (40)	0.353
Causes: Family history	15 (30)	42 (84)	38 (76)	0.001 *	15 (30)	15 (30)	16 (32)	0.969
Head trauma	16 (32)	41 (82)	34 (68)	0.001 *	16 (32)	17 (34)	17 (34)	0.97
Home environment	7 (14)	4 (8)	0 (0)	0.027 *	7 (14)	6 (12)	6 (12)	0.942
Watching excess TV and video games	2 (4)	6 (12)	0 (0)	0.025 *	2 (4)	4 (8)	5 (10)	0.503
Aggravating factors Watching excess TV and video games	19 (38)	39 (78)	25 (50)	0.001 *	20 (40)	22 (44)	23 (46)	0.827
Food that contain food additives and artificial colors	2 (4)	13 (26)	8 (16)	0.009 *	4 (8)	5 (10)	2 (4)	0.503
Family problems	16 (32)	31 (62)	21 (42)	0.009 *	18 (36)	15 (30)	16 (32)	0.809
Living with one parent	13 (26)	27 (54)	18 (36)	0.014 *	17 (34)	18 (36)	15 (30)	0.811
Treatment lines	31 (62)	42 (84)	37 (74)	0.045 *	25 (50)	32 (64)	39 (78)	0.014 *
Overall knowledge: Adequate	8 (16)	35 (70)	23 (46)	0.001 *	11 (22)	10 (20)	14 (28)	0.625

^a^ Friedman test, * statistically significant (*p* < 0.05).

**Table 2:** Multiple hierarchical logistic regression model for predictors of post-experimental retained knowledge regarding ADHD among teachers.

Predictors	B	S.E.	*p*-Value	OR (95% CI)		
Trial vs. control	2.59	0.54	0.001 *	13.31 (4.61–38.45)		
Less than 40 years old vs. above	0.04	0.14	0.746	1.05 (0.80–1.37)		
Non-Saudi vs. Saudi	0.20	0.70	0.776	1.22 (0.31–4.83)		
Postgraduate vs. university	1.45	0.74	0.04 *	4.27 (1.02–18.28)		
Above 10 years’ experience vs. below	−0.01	0.13	0.958	0.99 (0.76–1.29)		
Private school vs. public schools	0.38	0.51	0.459	1.46 (0.54–3.98)		
Constant	−6.55	4.33	0.131	0.00		
Model Calibration; Significance		77%; 0.002 *				
Model Discrimination (AUC)		0.81 ^a^				

OR = odds ratio, CI = Confidence interval, * Statistically significant (*p* < 0.05), AUC = Area Under the ROC curve, ^a^ = excellent discrimination.
